# Quantification of Bird-to-Bird and Bird-to-Human Infections during 2013 Novel H7N9 Avian Influenza Outbreak in China

**DOI:** 10.1371/journal.pone.0111834

**Published:** 2014-12-05

**Authors:** Ying-Hen Hsieh, Jianhong Wu, Jian Fang, Yong Yang, Jie Lou

**Affiliations:** 1 Department of Public Health, China Medical University, Taichung, Taiwan; 2 Center for Infectious Disease Education and Research, China Medical University, Taichung, Taiwan; 3 Department of Mathematics and Statistics, York University, Toronto, Ontario, Canada; 4 Centre for Disease Modelling, York University, Toronto, Ontario, Canada; 5 Department of Mathematics, Harbin Institute of Technology, Harbin, China; 6 Department of Mathematics, Shanghai University, Shanghai, China; Shanxi University, China

## Abstract

From February to May, 2013, 132 human avian influenza H7N9 cases were identified in China resulting in 37 deaths. We developed a novel, simple and effective compartmental modeling framework for transmissions among (wild and domestic) birds as well as from birds to human, to infer important epidemiological quantifiers, such as basic reproduction number for bird epidemic, bird-to-human infection rate and turning points of the epidemics, for the epidemic via human H7N9 case onset data and to acquire useful information regarding the bird-to-human transmission dynamics. Estimated basic reproduction number for infections among birds is 4.10 and the mean daily number of human infections per infected bird is 3.16*10^−5^ [3.08*10^−5^, 3.23*10^−5^]. The turning point of 2013 H7N9 epidemic is pinpointed at April 16 for bird infections and at April 9 for bird-to-human transmissions. Our result reveals very low level of bird-to-human infections, thus indicating minimal risk of widespread bird-to-human infections of H7N9 virus during the outbreak. Moreover, the turning point of the human epidemic, pinpointed at shortly after the implementation of full-scale control and intervention measures initiated in early April, further highlights the impact of timely actions on ending the outbreak. This is the first study where both the bird and human components of an avian influenza epidemic can be quantified using only the human case data.

## Introduction

The first human case of avian influenza A (H7N9) virus infection was initially identified in Shanghai, China in March 2013. 132 human cases had been identified by May 30, of which 37 resulted in death [1]. H7N9 infection in humans could cause severe illness with high mortality. Since October of 2013, sporadic cases continued to be reported in China and Hong Kong, SAR. Fortunately, there has been no evidence of sustained human-to-human transmission so far [2], with only one report of probable person-to-person transmission [3].

A major obstacle in the ascertainment of an avian influenza epidemic is that the evidence we observe, i.e., the human cases, is only the final outcome of the bird-human transmission process. That is, when human avian influenza cases such as the 1997 H5N1 and the 2013 H7N9 outbreaks occur, we know that there had been at the same time an epidemic among birds, but we generally are not able to observe it, especially in instances of avian influenza, where asymptomatic or mild avian diseases in wild birds and even domestic poultry occur routinely but are not closely monitored, or perhaps not even observable as in the case of wild birds. We only know it when it becomes human-transmissible. Therefore, the profound challenge is to attempt to infer information regarding the epidemic within the bird population from our observations of human infections and, subsequently, to obtain useful knowledge on the process of bird-to-human transmission.

In this work we propose a bird-human epidemic model with two parts to describe the dynamical relationship between the epidemic in bird population and the subsequent bird-to-human infections. The first part of the model is a modified SIR compartmental model adopted to describe the transmission among birds; the second is a simple single-equation model for the increase in the cumulative number of infected humans due to infection transmitted by infected birds. Our modeling approach allows us to infer important information regarding the bird epidemic via the model parameters for the bird population that are epidemiologically important. With these results, we can then acquire useful knowledge regarding the dynamics of bird-to-human transmissions. There have been numerous previous modeling studies on avian influenza epidemic of humans (e.g., [4]–[8]), or on the bird epidemic only [9]–[10]. Some theoretical studies also consider both the bird and human epidemics but mainly focusing on human infections [11]–[16]. Some recent studies on 2013 H7N9 have included both birds and human infections in their models, but with the purpose to quantify the human transmission parameters only (e.g., [14]–[16]). In our work, we are able to estimate the basic reproduction number for infections among birds and the mean number of human infections per infected bird per day. To our best knowledge, this is the first study where a bird-human avian influenza epidemic can be ascertained through the modeling of both the bird and human parts of the epidemic and, more importantly, quantified through the use of human case data only.

## Materials and Methods

### Data

We make use of the human H7N9 case data by onset date from February 19 to May 30, 2013 and made public by the World Health Organization (WHO) [1], to estimate the model parameter values, including the basic reproduction number [17] of the bird epidemic and final bird outbreak size. Note that 10 of the 132 confirmed cases during this time period did not have a date of onset; therefore the data we fitted contains 122 cases.

#### Transmission dynamics among birds

We stratify the total number (*N*(t)) of birds, “actually” vulnerable for the disease transmission at time *t*, into two classes: actually susceptible (*S*(t)) and infected (*I*(t)). Here we introduce the class of “actually at risk” susceptibles for birds that will be exposed to the virus during the entire epidemic under consideration (at “actual” risk for infection), which is consistent with the underlying idea of the Richards model [18] which we utilize in our modeling construction (see Mathematical Details section), where by definition only (eventually) infected population is considered. The introduction of this novel modeling construction is also critical for the purpose of its application to avian influenza modeling, since it is quite conceivable that a large number of birds (especially wild ones) were not exposed to the virus, but we do not have any information regarding its size. This definition of S(t) is different from the “susceptible” birds defined in the classical SIR model where (majority) susceptible birds may not be actually exposed to the virus. Therefore, the initial size of this actually at risk susceptible class is unknown and it is one of the parameters to be estimated. In what follows, we will use total bird population “actually at risk” at time t for N(t) and “actually” susceptibles at time t for S(t), but simply infected birds I(t) for sake of brevity. Detailed model description is given in the Mathematical Details section.

Assuming that the birth and natural death of birds are balanced during the disease outbreak period (that is, the bird population remains at a constant level if there is no disease), we have the Kermack-McKendrick model (see Mathematical Details section) to describe disease transmission in birds, with the density-dependent transmission rate (*β/N*), and the removal rate (*δ*). An important feature of our proposed model is that the “actually at risk” total bird population *N*(t) (again, we emphasize this is the total bird population to be involved in the disease transmission during the entire epidemic under consideration) can be determined, via a close form (analytic expression), from the initial numbers of the compartment sizes (*S*(0) = *S*_0_, *I*(0) = *I*_0_ and *N*(0) = *N*_0_) at the beginning of the bird infection outbreak and from the aforementioned parameters (see Mathematical Details section for details). Hence “removal” in our model refers to all removal from the actually infected bird population, including disease-induced death, recovery (although infected birds often exhibits only mild or no symptoms), or culling after April 5.

Since disease transmission is assumed to depend on the actually at risk total bird population size which varies in time, we define the time-varying reproduction number, or "running basic reproduction number" [19] for birds, 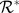 by
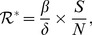
(1)which gives the number of secondary infections caused by a single infected bird in the population at time *t*. It is natural to assume that 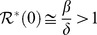, otherwise the disease will disappear quickly. Therefore, we have 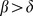.

We can further derive expressions for the cumulative number of infected bird population (also known as the bird outbreak size), the *peak time* of bird infections *t_p_*, which is shown to be equal to the turning point of infections among birds 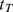 [20]–[28]. The detailed derivations are provided in Mathematical Details section.

#### Bird to human infection

**Figure 1:**
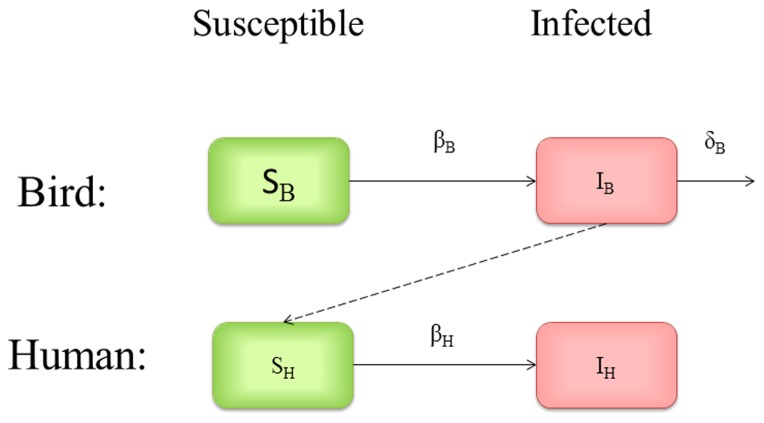
Model diagram for bird-human disease transmission model.

The human population susceptible to the H7N9 virus is assumed to remain at a constant level during the early stage of outbreak. Moreover, there is no evidence indicating that either human-to-human or human-to-bird infection had occurred. Thus, the cumulative number of human H7N9 cases by infected birds, *I_H_*, can be simply modeled (see Mathematical Details section) so the increase rate of the infected human individuals is proportional to the number of the infected birds, with the proportionality constant (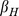)-the daily human infection rate per infected bird. Integration of this simple model establishes a link between the cumulative number of H7N9-infected humans and the total number of birds. See Mathematical Details section for more details. For illustration, the bird-human model diagram is provided in Figure 1.

#### Richards model

The Richards model growth function [18] given below
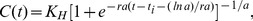
(2)describes the cumulative human case number at time t, where *K_H_* is the carrying capacity or final outbreak human case number of the human epidemic, *r* is the per capita growth rate of the cumulative case number, *a* is the exponent of deviation of the cumulative case curve, and *t_i_* is the turning point of the epidemic (which signifies the moment of upturn or downturn for the increase in the cumulative case number). We fit the human H7N9 case data to this Richards model growth function. We refer to [20] for the theoretical foundation for using Richards model to fit epidemic curves.

To model the initial stage of an outbreak, it is natural to assume that initially there are no recovered birds, i.e., *N*_0_ = *S*_0_+*I*_0_. Consequently, we may describe the bird-human epidemic, respectively, as a system of two coupled equations, one for the bird epidemic and the other for human outbreak. In this coupled system, there are five parameters to be estimated: *δ*, *β*, *N*_0_, *I_0_*, and *β_H_*. The first four parameters enable us to quantify the outbreak among bird population, while the last term is the bird-to-human infection rate.

To obtain estimates for the model parameters, we first fit the H7N9 human case data by onset date from February 19 to May 30, 2013, available from the WHO website [1], to the Richards model growth function given in Equation (2) where the time unit is in days. We then fit the human case data simultaneously to Equations (4) and (21) in the Mathematical Details section to obtain the 5 model parameters *δ*, *β*, *N*_0_, *I_0_*, and *β_H_*. Estimations are performed using the nonlinear least-squared subroutine in MATLAB.

## Results

### Epidemiological quantifiers

**Figure 2:**
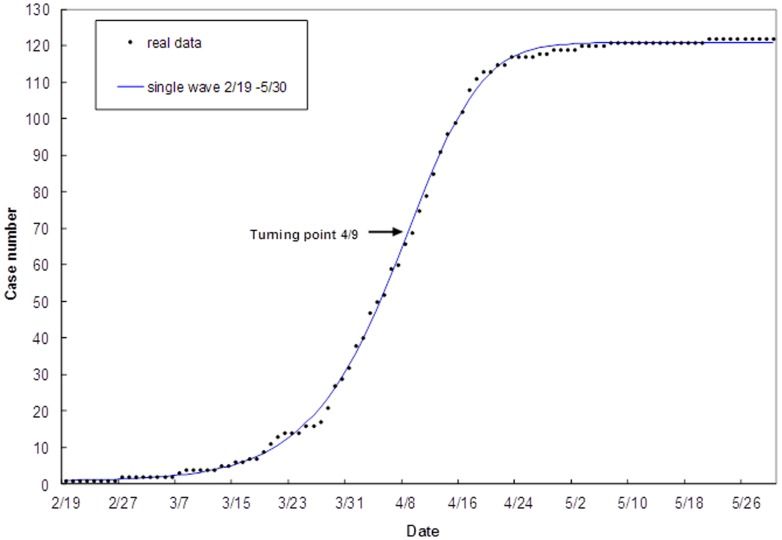
Model fit for cumulative human H7N9 case number in China, February 19-May 10, 2013 using the Richards model (2).

We first fit the human H7N9 case data to the cumulative human cases using the Richards model growth function (Equation (2)) to obtain model estimates for the turning point *t_i_* and the case number K_H_ of the Richards model growth function (see Figure 2 and Table 1). The turning point for the human case reporting, at 48.94 days (95% CI: [48.51, 49.37]) or the 49^th^ day after the start of outbreak on February 19, is pinpointed at April 9; while the estimate for the outbreak case number K_H_ is 120.4 [119.6, 121.2], exactly the same as the WHO data if rounded off to the next integer.

**Table 1:** Estimated model parameter values with 95% CIs (in brackets) via the bird-human epidemic model (Equations (4) and (21)) and the Richards model using H7N9 human case data by onset date in China, February 19––May 30, 2013.

Bird outbreak		Human outbreak	
Bird-to-bird infection rate *β*	0.3727 [0.3723,0.3731]	Bird-to-human infection rate *β_H_*	3.16*10^−5^ [3.08*10^−5^,3.23*10^−5^]
Initial total bird population actually at risk *N*_0_	764 477 [747981,780973]	Case number* K_H_	120.4 [119.6,121.2]
Initial number of infected birds *I_0_*	143.4 [142.7,144.1]	Turning point* *t_i_*	48.94 [48.51,49.37]
Removal rate *δ*	0.0909 [0.0906,0.0913]		
Turning point *t_T_*	55.96		
Basic reproduction number for birds 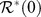	4.10		

Turning point for human outbreak on the 49^th^ day (48.94 days after February 19) implies occurring on April 9, while turning point for bird outbreak on the 56^th^ day (55.96 days after) pinpoints April 16.

*Estimated from human case data fitting with the Richards model growth function.

We then proceed to fit the same human H7N9 case data to the bird-human epidemic model, in order to obtain estimates for the removal rate *δ*, bird-to-bird infection rate *β*, initial actually at risk susceptible bird population size *N_0_*, initial infected bird population size *I_0_*, and bird-to-human infection rate *β_H_*. Least-squared fitting was performed using Matlab. Data fitting results with respective 95% confidence intervals (CI) are given in Table 1. From these estimates, we can further compute the turning point *t_T_* for the bird epidemic, the basic reproducing number for the H7N9 epidemic in the bird population 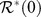, as well as the final outbreak size of the epidemic in the bird population via Equations (21)–(22) given in the Mathematical Details section. From the model fit for the bird-human epidemic model, we can also make use of the resulting parameter estimates to compute other epidemiologically meaningful parameter values. The turning point for the bird infections was 55.96 days (or the 56th day) after February 19, or April 16 (see Table 1).

## Discussion

We observe that the infections among birds began to turn for the better on the same day (April 9) as a similar downward turn occurring in the reporting of human cases. The estimated median incubation period in 23 confirmed H7N9 human cases for whom detailed data on animal and environmental exposures was reported to be 6 days (range: 1 to 10) [29]. Furthermore, a modeling study on 2013 H7N9 [14] proposes that the mean generation time of the bird-human transmission to be 2–4 days. Therefore, our result of turning point for reporting of human cases on April 9 indicates that the turning point for actual human infections had occurred 2–4 days before April 9 but most likely after April 5.

**Figure 3:**
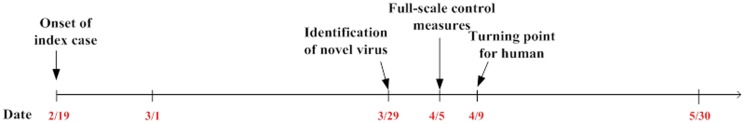
Chronological timeline of the human avian influenza H7N9 outbreak in China, February 19-May 10, 2013. April 9 is the turning point for bird-to-human infections as elucidated via the Richards model. April 16 is the turning point for bird epidemic concluded from the bird-human model.


It is interesting to note that on April 5, the Chinese Ministry of Health (MOH) announced full-scale measures to prevent spread of the novel virus infections [30]. In particular, the live poultry market in the affected areas was closed temporarily with culling of geese, chickens, ducks and pigeons, and spraying of disinfectant in the surrounding areas [31]. A total of 20,536 chickens, ducks, geese and pigeons from Huhuai Agricultural Products Wholesale Market in Shanghai alone were reported to have been slaughtered by April 5 after the H7N9 bird flu virus was detected from samples of pigeons in the market [32]. The swift responses after the identification of the novel virus on March 29 and the subsequent full-scale control measures after April 5 seemed to have a direct and almost immediate impact on human infections, in the following days, leading to a decrease in clinical cases around April 9. This may be attributable to swift human response and behavior change by the populace in reaction to government implementation of interventions measures. In comparison, the turning point for bird infections occurred one week later, on April 16. Since one could propose that much of the bird infections occurred among wild birds, it is reasonable to propose that culling of *domestic birds* and closing of live poultry farm did not have an immediate impact on infections among wild birds, but perhaps contributed to its turning point one week later. The chronological timeline of the bird-human H7N9 epidemic in China is illustrated in Figure 3.

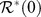
, the basic reproducing number for H7N9 epidemic *in bird population* or the secondary number of infections in birds by one new infected bird entering in a previously disease-free bird population, is found to be 4.10, showing considerable potential of the disease to spread among birds. In contrast, the exceedingly low bird-to-human daily infection rate *β_H_* of 0.0000316 seems to indicate that, even when an epidemic did occur in the bird population, the risk for human infection, whether directly from bird to human or indirectly through environmental contamination, is extremely low, assuming that there were no human-to-human transmission. Note that in this work we had assumed that there is neither human-to-human nor human-to-bird infection during the outbreak, since there is no evidence indicating such occurrences. If there had indeed been any human-to-human transmission of H7N9 virus occurring among the reported cases, as some studies had assumed [8], [14], inferred [7], or proposed [3], [15], *β_H_* would have been even lower to account for less bird-to-human infections. Moreover, for modeling consideration, if there had been human-to-human infections, then Equation (17) would need to be modified to account for human infections, perhaps with an added human susceptible class. This would lead to a far more complicated model with more parameters to estimate, e.g., human-to-human infection rate, and hence shall be left for future research.

We also note that culling measure implemented after April 5 was really a pre-emptive measure to remove the infective birds, and the susceptible birds from future exposure to infection. Therefore, this effect is indeed modeled in our setting since *S(0)* (or *S_0_*), the initial "at risk" population size, would be much bigger without this culling. It is important to note that *S(0)* is the initial population size of the birds which would be exposed to infection during the entire course of the epidemic, including the phase when culling was implemented.

In the affected Zhejiang-Jiangsu-Shanghai region, where there are over 170 million inhabitants and perhaps similarly large number of wild and domestic birds (if not significantly more if we also include migratory birds), 0.07% of the bird samples were found to be H7N9-positive by the Chinese Ministry of Agriculture [33]. Hence our estimate of total at-risk bird population that were eventually infected, N_0_, of 764 477 is quite reasonable. However, only 132 cases (less than 1 in one million) were diagnosed during that time. This indicates a wide-spread bird epidemic but significantly smaller human epidemic. This is consistent with our conclusion.

The main difficulty in ascertaining avian influenza outbreak in humans, especially one where majority of the cases were caused by bird-to-human infections, comes from the fact that very little is known of the bird epidemic; e.g., the bird population size, the number of infected birds, types of birds that are being infected, and the composition of the birds (domestic/poultry birds, local wild birds, migratory birds, etc.) Consequently, the key contribution and novelty of our work is that we are able to extract important information regarding the epidemiology (including bird-to-bird basic reproduction number and bird-to-human infection rate) of avian influenza epidemic among birds via our modeling using only human case data, which is important to our understanding and efforts for prevention of avian influenza outbreaks that includes mitigation of bird epidemic.

Our estimates of the basic reproduction number for the bird outbreak and the bird-to-human daily infection rate suggest that, even with the wide-spread H7N9 infections in bird population, the threat to human population is currently not particularly severe and can be effectively averted through swift and efficient control measures. Although the resulting high mortality of the infected persons requires our most diligent efforts to prevent as much as possible the occurrence of loss in lives, and perhaps more importantly, to continue monitoring any sign of future emergence and escalation in H7N9 human cases in case a human-to-human transmissible mutation occurs in the virus, especially as more cases of human H7N9 cases have been reported since the fall of 2013, in the Zhejiang-Jiangsu-Shanghai region and many other provinces along the southeast coast of China [34].

A limitation of our study is the fact that ten of the human cases during the time interval under study have no date of onset and therefore were deleted from the fitted data, which might lead to some estimation error. However, the number of cases deleted is less than 10% of the dataset and hence is not likely cause a major discrepancy in the qualitative results. As a final note, our model does not incorporate control measures explicitly, which requires a far more complicated model, and much more detailed data typically difficult to gather. However, we suggest that the timing of the turning points obtained through our modeling might reflect the impact of the control measures implemented. For this H7N9 epidemic with specific routes of transmission and limited infections, a network model might be a useful tool for inferring the impact of culling as well as the closing of poultry market [16]. However, for such an endeavor substantially more detailed data are critically needed to carry out such a modeling study, namely, complete information on the social networking of the infected individuals and, for avian influenza outbreak, their exposure-related activities (with poultry, domestic and wild birds, and environment) that potentially might have led to their infections, which unfortunately are not readily available.

## Mathematical Details

### Kermack-McKendrick model with actually at risk (for infection) bird population

We adopt the classical Kermack-McKendrick SIR model to describe disease transmission in birds:
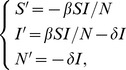
(3)where the prime means derivative with respect to time 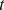, *β/N* is the density-dependent transmission rate, and *δ* is the removal rate (including disease-induced death and culling) Recall that S is the size of the “actually” susceptible bird population.

According to [20], we may see that the initial value problem of system (3) can be decoupled. More precisely, the equation that determines the total bird population (actually at risk for infection) at time t is
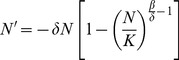
(4)with
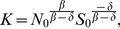
(5)which is close to 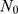 if 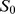 is close to 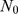. Here the subscript “0” denotes the variable at time t = 0. Moreover, *S* and *I* can be explicitly expressed as functions of *N* by
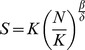
(6)and
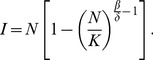
(7)


### Epidemic characteristics of bird infection

It is natural to assume that 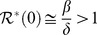, otherwise the disease will disappear quickly. Therefore, we have 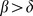. Meanwhile, a direct calculation with the aid of (6) yields
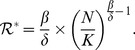
(8)


Consequently,
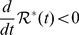
(9)since 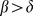.

Let the subscripted infinity “*∞*” denote the limit as *t→∞*. Then the cumulative number of infected population, also known as the *outbreak size*, can be determined by the quantity 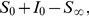
(10)which is shown to coincide with
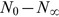
(11)using the fact *I_∞_ = 0*. Thus, to investigate the outbreak size, it suffices to analyze the final size of total population *N*_∞_, which is the unique positive solution to the following equation with unknown *x*:
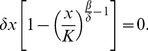
(12)


Therefore, 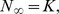 and hence, the outbreak size equals to 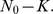 At the *peak time* of bird infection *t_p_* [20], we have 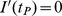, which is equivalent to 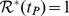. Hence, from the expression for 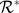 in (8) we have
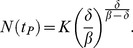
(13)


Consequently, 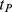 can be computed from the equation for *N*. Moreover, combining the expression for 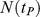, and for *I* in (7), we obtain the peak size of bird infections
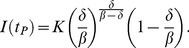
(14)


At the turning point of infections among birds 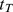 [20]–[28], we have 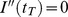. Note that
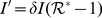
(15)


It then follows that 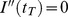 is equivalent to
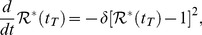
(16)from which we can compute 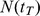 by making use of Equations (4–6) and (8). Subsequently, the turning point 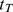 can also be computed by equation of *N*.

### Model for human infection

The cumulative number of human H7N9 cases by infected birds, *I_H_*, can be modeled simply by
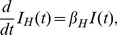
(17)where 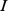 denotes the infected bird population at time t and 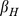 is the daily number of human infection per infected bird. Note also that our model for human epidemic (17) considers only those with onset (as the human case data is by onset date), which is reasonable if we assume that, for H7N9, humans have no infectivity before onset of symptoms.

We can solve (17) to obtain
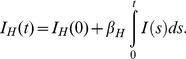
(18)


Recall that System (3) is used to model the bird infection. Then the total bird population 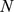 has the following relation with the infected bird population 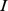:
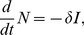
(19)which implies
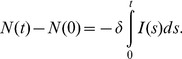
(20)


Hence,
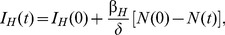
(21)which establishes a link between the cumulative number of H7N9-infected humans and the total number of birds.

Finally, we note that
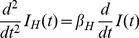
(22)


Therefore, we conclude that the turning point of the cumulative number of reported human cases also coincides with the peak time of bird infections.
